# Identification of QTL conferring resistance to stripe rust (*Puccinia striiformis* f. sp. *hordei*) and leaf rust (*Puccinia hordei*) in barley using nested association mapping (NAM)

**DOI:** 10.1371/journal.pone.0191666

**Published:** 2018-01-25

**Authors:** Thomas Vatter, Andreas Maurer, Dragan Perovic, Doris Kopahnke, Klaus Pillen, Frank Ordon

**Affiliations:** 1 Federal Research Centre for Cultivated Plants, Institute for Resistance Research and Stress Tolerance, Julius Kuehn-Institute (JKI), Quedlinburg, Germany; 2 Martin Luther University Halle-Wittenberg, Institute of Agricultural and Nutritional Sciences, Chair of Plant Breeding, Halle, Germany; Western Australia Department of Agriculture and Food, AUSTRALIA

## Abstract

The biotrophic rust fungi *Puccinia hordei* and *Puccinia striiformis* are important barley pathogens with the potential to cause high yield losses through an epidemic spread. The identification of QTL conferring resistance to these pathogens is the basis for targeted breeding approaches aiming to improve stripe rust and leaf rust resistance of modern cultivars. Exploiting the allelic richness of wild barley accessions proved to be a valuable tool to broaden the genetic base of resistance of barley cultivars. In this study, SNP-based nested association mapping (NAM) was performed to map stripe rust and leaf rust resistance QTL in the barley NAM population HEB-25, comprising 1,420 lines derived from BC_1_S_3_ generation. By scoring the percentage of infected leaf area, followed by calculation of the area under the disease progress curve and the average ordinate during a two-year field trial, a large variability of resistance across and within HEB-25 families was observed. NAM based on 5,715 informative SNPs resulted in the identification of twelve and eleven robust QTL for resistance against stripe rust and leaf rust, respectively. Out of these, eight QTL for stripe rust and two QTL for leaf rust are considered novel showing no overlap with previously reported resistance QTL. Overall, resistance to both pathogens in HEB-25 is most likely due to the accumulation of numerous small effect loci. In addition, the NAM results indicate that the 25 wild donor QTL alleles present in HEB-25 strongly differ in regard to their individual effect on rust resistance. In future, the NAM concept will allow to select and combine individual wild barley alleles from different HEB parents to increase rust resistance in barley. The HEB-25 results will support to unravel the genetic basis of rust resistance in barley, and to improve resistance against stripe rust and leaf rust of modern barley cultivars.

## Introduction

The biotrophic rust fungi *Puccinia hordei (Ph)*, causing leaf rust, and *Puccinia striiformis* f. sp. *hordei*, *(Psh)*, the causal agent of stripe rust, are important barley pathogens in many barley growing areas worldwide [1, 2]. The ability of the two rust fungi to spread across large distances, rapidly increase in population size, and mutate quickly [3–5] results in a high risk for severe epidemics. Infection can cause yield losses of up to 62% in case of *Ph* and up to 70% in case of *Psh* and reduce grain quality under epidemic conditions [6–8]. Depending on environmental conditions, there is a high variability in disease severity between years. While *Psh* in general has been of minor economic importance over the last several decades the importance of *Ph* has increased [4, 5, 9–11]. Nevertheless *Psh* remains a major economic threat, especially for barley production in Australia where it is not yet present, as studies in Mexico identified that 70% of Australian barley varieties are susceptible to the aggressive *Psh* race 24 [12, 13].

Although both fungi can be controlled by timely fungicide application, emphasis should be laid on resistance breeding as a cost effective, environmental, and consumer-friendly alternative [4, 14, 15]. Most promising in this regard is to breed for cultivars exhibiting both race-specific and non-race specific resistances [16].

Up to now 25 major genes (*Rph1*-*Rph24*) and *Rph*_MBR1012_ conferring resistance to *Ph* have been reported of which all but one were assigned to chromosome regions [5, 17, 18]. Out of these, *Rph15*, *Rph16*, and *Rph*_MBR1012_ [17, 19, 20] are still effective in Europe, whereas in Israel, Morocco, Spain, and the USA *Rph7* has already been overcome by new *Ph* races [21–24]. Given the ability of the fungus to spread across large distances and to mutate quickly, it is only a matter of time until still effective *Rph* genes will be overcome as well. The identification of race non-specific quantitative resistance and its introgression into modern barley cultivars is therefore of highest importance. Numerous studies focusing on resistance of barley to *Ph* resulted in the identification of a high number of QTL located on all barley chromosomes [25–39].

Over the last several decades less research has been conducted on resistance of barley to *Psh* due to its significantly lower importance compared to *Ph*. However, 26 uniquely different *Rps* (*Resistance to Puccinia striiformis*) major genes (reviewed in [40]) and several QTL [11, 38, 41–53] have been reported up to now.

In almost all studies focusing on the identification of QTL and genes conferring resistance to *Ph* or *Psh* bi-parental linkage mapping (LM) was applied. Association mapping (AM) to detect resistance QTL was, to our best knowledge, applied in only one study for resistance to *Psh* [44] and in two studies for resistance to *Ph* [35, 36]. Furthermore, Schnaithmann et al. [25] applied a nested association mapping (NAM) approach based on an explorative multi-parental NAM population to detect QTL conferring seedling resistance to *Ph*. A large-scale NAM study based on field trials to identify resistance QTL has not been conducted yet for either of the two fungi.

NAM is based on a multi-parental mapping design introduced by Yu et al. [54] as a genome-wide association strategy to dissect the genetics of complex traits. NAM combines the advantages of conventional LM and AM strategies, namely the high detection power per SNP and the high allelic richness, allowing for an exceptional high mapping resolution [54–56]. Next to several studies based on the initial maize NAM population [54, 55, 57–64], NAM studies were conducted in a second maize NAM population [65], sorghum [66], wheat [67, 68] and barley [25, 69–76] highlighting the power of this mapping approach.

Until now, the world’s first barley NAM population introduced by Maurer et al. [69] named ‘Halle Exotic Barley 25’ (HEB-25) has not been used to identify QTL linked to resistance to biotrophic fungi. Thus, in this study the genetic diversity present in HEB-25 combined with the exceptional high mapping resolution offered by NAM was used to achieve the following objectives: I) to screen the HEB-25 population for resistance against *Ph and Psh*; II) to identify QTL conferring resistance against *Ph* and *Psh*; III) to identify HEB-25 lines with strong resistance, suitable to be introduced in pre-breeding programs; IV) to compare QTL positions detected in this study with those previously reported in literature, and V) to identify putative candidate genes underlying the identified resistance QTL.

## Material and methods

### Plant material

This study is based on the HEB-25 NAM population [69]. HEB-25 comprises 1,420 BC_1_S_3_ lines in 25 families, originating from crossing 25 highly divergent wild barley accessions (*Hordeum vulgare* ssp. *spontaneum* and *H*. *agriocrithon*) with the modern spring barley cultivar Barke (*Hordeum vulgare* ssp. *vulgare*). For more detailed information on population development see Maurer et al. [69]. Due to a loss of genotypes during field trials, the analysis is based on 1,401 genotypes of the HEB-25 population.

### Field trials

Field trials were conducted at the Julius Kuehn-Institute, Federal Research Centre for Cultivated Plants, in Quedlinburg, Germany, during the seasons 2014 and 2015 using a randomized incomplete block design with two replications. Screening for resistance to *Ph* and *Psh* was performed in separate field trials. Genotypes were sown in double rows of 1 m length with 25 plants per row and spacing of 0.2 m between rows in mid-March in both years. Incomplete blocks were surrounded by spreader strips of susceptible varieties. Spreader strips were spray inoculated with an oil-spore mixture using a hand-held spinning disc sprayer (ULVA+, Micron Sprayers, Bromyard, Herefordshire, U.K.) to ensure homogeneous disease pressure. A 1:1 mixing ratio of rust spores in mg to oil in ml (Isopar M, ExxonMobil Chemical Company, Spring, TX, USA) and 100 ml of suspension per 30 m^2^ was used for inoculation. Starting at shooting, spray inoculation was performed at three dates early in the morning when dew formation was observed. For leaf rust (*Ph*) trials isolate I-80 was used, a very destructive leaf rust isolate overcoming common major resistance genes in the European barley gene pool, except *Rph7*, *Rph15*, *Rph16*, and *Rph*_*MBR1012*_ [17, 19, 20]. The virulence of I-80 against *Rph17*–*Rph24* has not been surveyed yet. For stripe rust (*Psh*) trials, the very aggressive race R-24 was used, which is wildly spread in Europe and the Americas [4, 7, 53].

### Phenotypic data

Percentage of infected leaf area (PILA) was recorded at three subsequent dates according to Moll et al. [77], starting when disease symptoms were clearly visible in the susceptible spreader strips. A time period of two weeks between phenotyping dates was chosen to allow for sufficient disease development. Based on PILA data the area under the disease progress curve (AUDPC) was calculated for each genotype. AUDPC data were then used to calculate the average ordinate (AO [78]) for each genotype as a measure of infection severity:

$$AO=\frac{\overset{N_{i-1}}{\underset{i=1}{\sum }}\frac{\left(y_{i}+y_{i+1}\right)}{2}*\left(t_{i+1}-t_{i}\right)}{tp}$$

where (*N*) is the total number of observations, disease level at the ith observation is coded by (*y*_*i*_), time at the ith observation is coded by (*t*_*i*_), and the total trial period in days is coded by (*tp*).

### Statistical analysis

Phenotypic data analysis was performed using the software package SAS 9.4 (SAS Institute Inc., Cary, NC, USA) using *proc mixed*. Genotype, year, and genotype *x* year interaction were set as fixed effects. Design effects were set as random statement. Separate co-variances were set for years to account for the difference in disease pressure between years. To meet the requirements of mixed linear model analysis, phenotypic raw data was log_10_ transformed before applying the mixed procedure. Obtained AO log_10_ least squares means (lsmeans) were used for subsequent nested association mapping (NAM). To estimate variance components to be used for the calculation of broad sense heritability (h^2^) all model parameters were set as random. Broad sense heritability across years was calculated as:

$$h^{2}=\frac{V_{G}}{V_{G}+\frac{V_{GY}}{y}+\frac{V_{R}}{yr}}$$

where genotypic variance is coded by (*V*_*G*_), genotype *x* year variance is coded by (*V*_*GY*_), and residual variance is coded by (*V*_*R*_). The terms *y* and *r* represent the number of years and replicates, respectively.

Pearson’s correlation coefficients were calculated with *proc corr*, using lsmeans per genotype as input.

### Nested association mapping

SNP genotyping was carried out using the barley Infinium iSelect 9K chip consisting of 7,864 SNPs [79]. SNPs showing >10% failure rate, >12.5% heterozygous calls, or being monomorphic over all 1,401 HEB lines were removed from the dataset. SNP filtering resulted in 5,715 informative SNPs used for NAM with an average genetic distance of 0.17 cM and a maximum gap of 11.1 cM between adjacent markers. Linkage disequilibrium (LD) across HEB-25 was calculated as r^2^ between all mapped SNPs, excluding heterozygous genotypes, with the software package TASSEL 5.0 [80]. LD decay across intra-chromosomal SNPs was displayed by plotting r^2^ between SNP pairs against their genetic distance. A second-degree smoothed loess curve was fitted in SAS with *proc loess*. The population-specific baseline r^2^ was defined as the 95^th^ percentile of the distribution of r^2^ for unlinked markers [81]. LD decay was defined as the distance at which the loess curve crosses the baseline. An identity-by-state approach was used to differentiate HEB genotypes. Parental genotype information enabled the identification of the exotic donor allele in each segregating HEB family. HEB lines showing a homozygous Barke genotype were assigned a value of 0, HEB lines showing a homozygous exotic genotype were assigned a value of 2, and heterozygous HEB lines were assigned a value of 1. Failed SNP calls were assigned a value using the mean imputation (MNI) approach [82]. For detailed information see Maurer et al. (2015). Assignment of SNPs to chromosomal positions was based on Comadran et al. [79] and Maurer et al. [69].

NAM was performed using Model B of Liu et al. [83] verified to be best suited for genome-wide association studies (GWAS) based on family-structured populations [84] and successfully applied in previous HEB-25 studies [69–71]. Model B is a multiple regression model including, next to a quantitative SNP effect and a qualitative family effect, quantitative cofactors that correct for population stratification and genetic background noise [84]. Marker trait associations were estimated by stepwise forward-backward regression based on minimizing the Bayesian information criterion (BIC [85]) taking into consideration all informative SNPs. Analysis was carried out with SAS 9.4 applying the *proc glmselect* procedure. SNPs were allowed to enter or leave the model at each step until the BIC estimate was not reduced any further. SNPs included in the final model were defined to be significant.

To increase the robustness of identified marker trait associations, a five-fold cross-validation (CV) was performed. In total, 200 CV runs (40 times five-fold CV) were performed. For this, 200 subsets were extracted out of the full genotype set. Each subset included 80% of genotypes of the full population, randomly selected per HEB family. The subsets were taken as training sets for the identification of significant marker trait associations and for estimation of additive effects. The remaining 20% of genotypes were used as the validation set. Subsequently, the count of each significant marker over all training sets was recorded and referred to as detection rate (DR). This value was taken as a measure of robustness of the marker trait association. Markers with a DR of >50% were defined as robust and used to assign resistance QTL.

Additive effects for each SNP were extracted as regression coefficient of the respective SNP directly from the NAM model described above. To obtain final estimates, additive effects of significant markers were averaged across all runs. Likewise, final R^2^ values for significant SNPs were obtained by averaging R^2^ values of significant markers across all cross-validation runs. This way, the R^2^ value can be interpreted as the percentage of variance explained by the investigated SNP marker.

A standard QTL interval of ±4 cM around the markers with a DR >50% was defined, representing the LD decay in HEB-25 (S1 File). In case the QTL was composed of more than one marker with a DR >50%, the marker showing the highest DR across all 200 cross-validation runs was defined as peak marker. QTL showing overlapping QTL intervals were combined to a single QTL interval.

To estimate the proportion of phenotypic variance explained by the full model, the unbiased estimator R^2^_adj_ [86] was calculated for each subset by simultaneously modeling all of the significant markers in the linear model described above.

To determine the predictive ability R^2^_pred_ of the full model for infection severity, the additive effects of markers estimated using the training sets were used to predict the phenotypic value of the remaining 20% of genotypes forming the validation sets [87]. Following Maurer et al. [70] R^2^_pred_ was defined to be the squared Pearson product-moment correlation between predicted and observed phenotypic values. Subsequently, R^2^_adj_ and R^2^_pred_ values were averaged over all 200 CV runs to obtain final estimates.

Additional to the detection of marker trait associations across families, parent-specific QTL effects were calculated following the approach of Maurer et al. [72]. In a first step, the peak marker (SNP with highest DR >50% across all 200 cross-validation runs) of each QTL was selected and placed central in a 26cM interval (obtained through simulation studies and representing the mean introgression size in HEB-25) to look for significant SNPs in this region. Due to model limitations reported in Maurer et al. [72] population-wide QTL located within this interval were pooled into one single parent-specific QTL. Subsequently, ‘Model-B’ SNP effect estimates of all markers within this interval were cumulated for each of the 25 donors, following $\overset{n}{\underset{i}{\sum }}{SNP\;(donor)}_{i}{*\alpha }_{i}$, where (*i*) iterates through all significant SNPs (*n*) in the respective QTL interval. *SNP* (*donor*)*_i_* represents the quantitative IBS donor genotype (i. e. 0 vs. 2) of the ith significant SNP and *α_i_* denotes the SNP effect estimate of this SNP obtained from ‘Model-B’. Since SNPs show different IBS segregation patterns across the donors of HEB families a different cumulated effect was obtained for each donor. This procedure was conducted within each of the 200 cross-validation runs. Subsequently, the mean effect across all cross-validation runs was calculated and taken as the final parent-specific QTL effect estimate.

### Comparison with previously identified QTL and analysis of identified QTL intervals

GrainGenes (https://wheat.pw.usda.gov/GG3/) and IPK Gatersleben (http://www.ipk-gatersleben.de/datenbanken/) databases were used to obtain marker information of previously reported QTL for *Ph* and *Psh* resistance. If available, this information was used to check for overlap of resistance QTL identified in this study with those already reported. The BARLEYMAP pipeline [88] was used as a common reference. Using this pipeline, the peak marker as well as flanking markers for known *Ph* and *Psh* resistance QTL and markers identified in this study showing a DR >50% were blasted against the POPSEQ map [89] and the barley physical map [90]. Markers with a DR >50% identified in this study and located in a genetic distance of less than 4 cM (representing the LD decay in HEB-25, see S1 File) to markers of known resistance QTL were defined as potentially corresponding to previously reported resistance QTL. In addition, previously reported QTL, for which no marker information could be obtained, were compared to QTL detected in this study based on information given in the respective publication.

In addition, the BARLEYMAP pipeline [88] was used to identify potential candidate genes underlying the robust QTL of this study by aligning the associated markers showing a DR >50% against the barley physical map [90] and the POPSEQ map [89]. The gene search was extended to an interval of ±4 cM around markers with a DR >50% to account for the LD decay in HEB-25. Gene ontology (GO) terms defining defence response (0006952, 0050832), apoptotic process (0006915), peroxidase activity (0004601), response to (oxidative) stress (0006979, 0006950), ATP binding (0005524), nucleotide binding (0000166), protein binding (0005515), transporter activity (0005215), and protein kinase activity (004672) were used to validate genes involved in resistance reactions [91]. Furthermore, GO terms defining reactions potentially involved, e.g. catalase activity, chitinase activity, cell wall peroxisome, cell wall modification, and defence response to fungi, were considered too (S2 File).

## Results

### Phenotypic analysis

**Fig 1:**
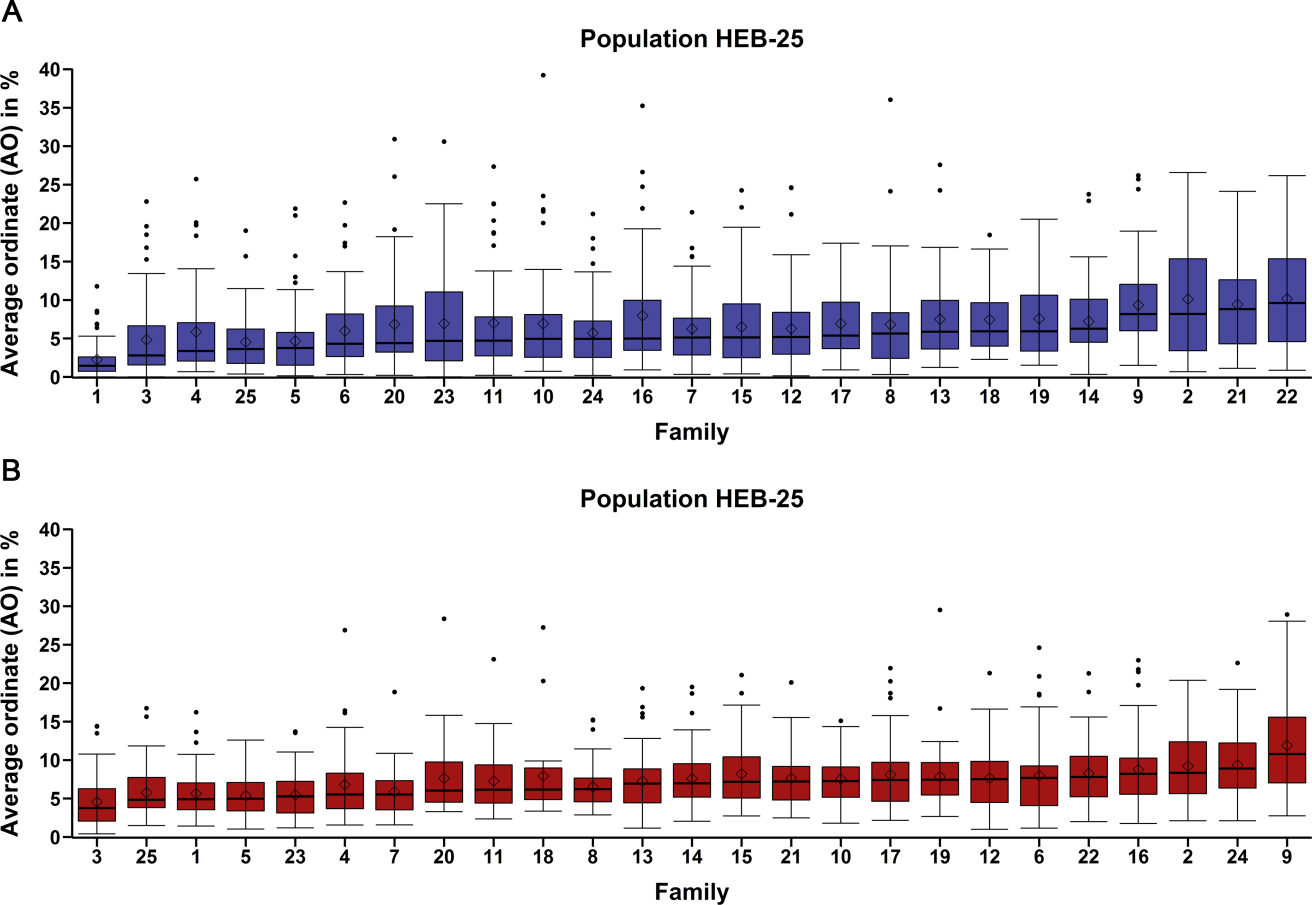
Box-whisker plots per HEB family indicating the variation in genotype response to the two fungi. **(A)** stripe rust (*Psh*) and **(B)** leaf rust (*Ph*) infection. HEB-25 families (1–25), sorted by ascending median, and rust severity are depicted on x-axis and y-axis, respectively.

Artificial infection resulted in a moderate disease pressure in both years, despite dry weather conditions impeding the initial infection process in the beginning of field trials. Nevertheless, experimental conditions allowed for an unequivocal scoring of resistance to *Psh* and *Ph*. A large variation concerning resistance was detected in the HEB-25 population for both pathogens. Significant differences (p <0.0001; Tukey-test) were observed between as well as within families in both cases (Fig 1A and 1B; S3 File). HEB families 1, 3, and 25 showed the highest resistance to both pathogens based on the AO median.

AO values ranged from 0% up to 39.2% in case of *Psh* and from 0.4% up to 29.5% in case of *Ph* (Table 1). AO frequency distributions of the HEB-25 population showed to be highly right skewed for both rust fungi, with *Psh* results showing slightly stronger skewness (S4 File). For *Ph*, cultivar Barke showed an intermediate degree of resistance compared to the wild donor parents, whereas in case of *Psh* the common parent Barke showed a very high degree of resistance.

**Table 1:** Descriptive statistics for two-year field trials in Quedlinburg and heritability.

Trait^a^	N^b^	Mean Barke^c^	Mean HEB-25^d^	Min^e^	Max^f^	SE_+/-_^g^	CV^h^	h^2^^i^
AO_*Psh*_	1401	3.31	6.72	0	39.23	0.15	0.85	0.70
AO_*Ph*_	1401	10.97	7.36	0.40	29.52	0.11	0.58	0.60

^a^Average ordinate for stripe rust (AO_*Psh*_) and leaf rust (AO_*Ph*_), respectively.

^b^Number of genotypes analyzed.

^c^Mean average ordinate of recurrent parent Barke.

^d^Mean average ordinate of the HEB-25 population.

^e^Minimum.

^f^Maximum.

^g^Standard error.

^h^Coefficient of variation.

^i^Broad-sense heritability.

In most cases, a higher susceptibility of wild accessions to *Psh* than *Ph* was observed. The wild donor of family 25 showed, among all wild donors, the highest resistance to both pathogens (S5 File). In general, only a weak correlation (Pearson’s correlation coefficients; p <0.0001; Tukey-test) of r = 0.28 between *Psh* and *Ph* infection of genotypes was identified across the whole HEB-25 population. Two-year broad sense heritability was calculated as h^2^ = 0.70 for *Psh* and h^2^ = 0.60 for *Ph* (Table 1).

### Nested association mapping

**Fig 2:**
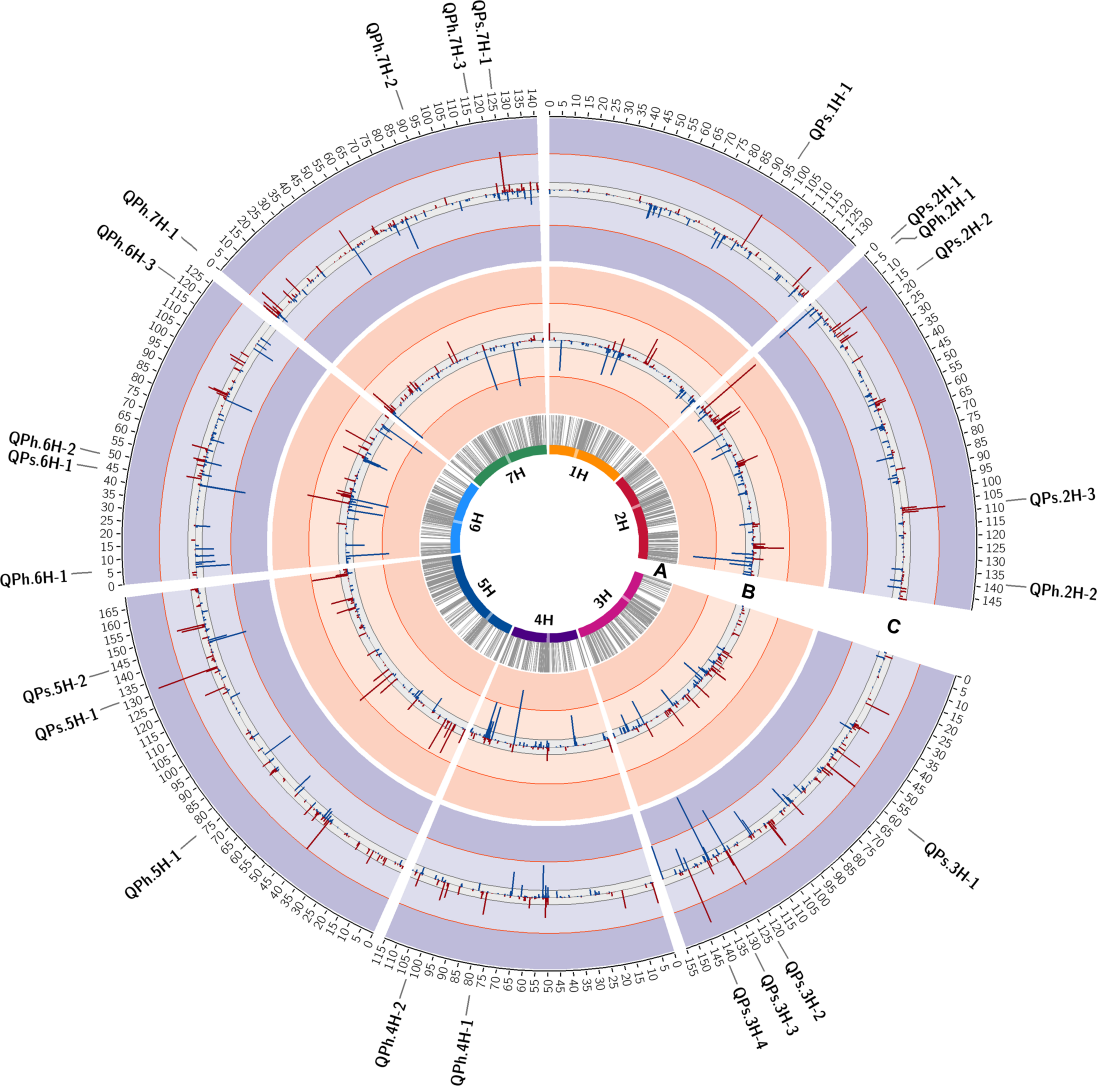
Circos plot indicating QTL controlling stripe rust and leaf rust resistance across HEB families. The barley chromosomes are arranged as coloured bars forming the most inner circle. Centromere regions are highlighted as transparent boxes. **(A)** Grey connector lines represent the genetic position of the 5,715 informative SNPs on the chromosomes with cM positions (based on Maurer et al. 2015) given on the scale outside of circle C. **(B)** Marker trait associations calculated for leaf rust data (AO_*Ph*_). Bars identify the position and detection rate (DR, height of bars) of significant marker trait associations. Bars in blue, pointing inwards, indicate a population wide trait-decreasing effect exerted by the wild barley allele, whereas bars in red, pointing outwards, indicate a population wide trait-increasing effect exerted by the wild barley allele. The grey and orange lines depict the DR threshold of 10% and 50% across 200 cross-validation runs. **(C)** Marker trait associations calculated for stripe rust data (AO_*Psh*_). Graphical representation are the same as described under (A). The position of the 23 robust QTL with DR >50% are indicated on the scale outside of circle C. QTL for stripe rust and leaf rust resistance are coded with QPs and QPh, respectively.

NAM was performed separately for each trait and resulted in the identification of numerous marker trait associations across HEB families (Fig 2; S6 File). However, most of the marker trait associations showed a DR below 50%. NAM based on *Psh* data resulted in the identification of 12 robust resistance QTL being composed of one or more markers with a DR higher than 50%, whereas NAM based on *Ph* data allowed to identify 11 robust resistance QTL (Table 2). For *Psh* QTL were identified on all chromosomes except chromosome 4H, whereas for *Ph* resistance QTL were identified on all chromosomes except chromosomes 1H and 3H (Table 2). Results of the two NAM studies showed that in most cases resistance QTL for *Ph* and *Psh* map to different chromosome regions. However, three chromosome regions were identified where *Psh* and *Ph* resistance QTL co-locate (Fig 2, Table 2). In detail, co-localisation of resistance QTL was observed on the short arm of chromosome 2H (QPs.2H-1; QPh.2H-1), within the centromeric region of chromosome 6H (QPs.6H-1; QPh.6H-2), and on the long arm of chromosome 7H (QPs.7H-1; QPh.7H-3).

**Table 2:** Robust stripe rust and leaf rust resistance QTL in HEB-25, detected with DR >50%.

QTL	Chr^a^	Peak marker with DR >50%^b^	Position of peakmarker (cM)^c^	DR in 200 CV runs (%)^d^	CV mean R^2^ (%)^e^	CV mean effect^f^	Corresponding resistance QTL/genes^g^
**Stripe rust(*Psh*)**							
QPs.1H-1	1H	i_SCRI_RS_136856	95.6	52.5	0.53	+0.16 (+1.45)	
QPs.2H-1	2H	i_SCRI_RS_165171	2.0	63.0	0.54	-0.34 (-2.19)	
QPs.2H-2	2H	i_SCRI_RS_159228	16.8	58.5	4.39	+0.13 (+1.35)	
QPs.2H-3	2H	i_SCRI_RS_158091	107.9	60.5	0.68	+0.31 (+2.04)	
QPs.3H-1	3H	i_12_30616	59.6	50.5	2.01	+0.13 (+1.35)	
QPs.3H-2	3H	**i_11_20146** i_SCRI_RS_235770	122.3	65.0 55.0	4.25	-0.22 (-1.66)	QTL_Toojinda[48] QTL_Yan/Chen[50]
QPs.3H-3	3H	i_SCRI_RS_209285	131.7	98.0	5.61	-0.28 (-1.91)	
QPs.3H-4	3H	i_12_20198	142.1	80.5	0.27	+0.41 (+2.57)	
QPs.5H-1	5H	i_SCRI_RS_175848	131.7	87.0	12.91	+0.31 (+2.04)	QTL_Cakir[52]
QPs.5H-2	5H	i_SCRI_RS_138608	143.8	53.5	0.09	-0.35 (-2.24)	
QPs.6H-1	6H	**i_SCRI_RS_162771** i_SCRI_RS_196285	43.7	64.0 60.5	2.73	-0.29 (-1.95)	QTL_Hayes[49] QTL6[42] QTL_Toojinda[48]
QPs.7H-1	7H	i_SCRI_RS_220680	125.1	57.0	0.86	+0.13 (+1.35)	*RpsFra*[44] *Rpsx*[43]
**Leaf rust (*Ph*)**							
QPh.2H-1	2H	i_SCRI_RS_184395	4.5	94.5	6.00	+0.12 (+1.32)	RphQ5[36] *Rph17*[92]
QPh.2H-2	2H	i_SCRI_RS_154135	138.6	81.0	5.48	-0.19 (-1.55)	QRph.sun-2H.2[37]
QPh.4H-1	4H	i_11_20670	78.4	75.0	7.67	-0.15 (-1.41)	Rphq5[26] RphQ8[36] QTL_Hickey[35]
QPh.4H-2	4H	i_SCRI_RS_148773	102.2	50.5	1.00	-0.28 (-1.91)	*Rph21*[93] QLr.S42-4H.a[32]
QPh.5H-1	5H	i_SCRI_RS_212784	75.6	57.5	4.05	+0.08 (+1.20)	
QPh.6H-1	6H	i_11_20882	5.6	59.0	1.26	-0.24 (-1.74)	QTL_Castro[34] QTL_Rossi[38]
QPh.6H-2	6H	**i_SCRI_RS_128460** i_SCRI_RS_128181	49.1	58.5 52.5	3.29	+0.30 (+2.00)	Rphq3[26] QTL_Castro[34] RphQ11[36] QTL_Hickey[35] *Rph24*[18]
QPh.6H-3	6H	i_11_11488	118.6	65.0	0.08	-0.10 (-1.26)	QTL_Backes[31]
QPh.7H-1	7H	i_SCRI_RS_208186	0.2	53.0	0.30	-0.26 (-1.82)	RphQ12[36]
QPh.7H-2	7H	i_12_20611	91.9	56.5	2.14	-0.08 (-1.20)	
QPh.7H-3	7H	i_SCRI_RS_175568	116.1	59.0	2.41	-0.07 (-1.17)	Rphq9[26] QTL_Castro[34]

^a^Chromosomal location of QTL.

^b^ISelect name of peak marker with a detection rate (DR) >50%. In case a QTL is composed of several SNP markers, the peak marker with highest DR is shown in bold letters.

^c^Position of the QTL peak marker based on Maurer et al. (2015).

^d^DR of the QTL peak marker in 200 cross-validation runs in percent.

^e^Mean percentage of phenotypic variance explained by the QTL peak marker, based on 200 cross-validation runs.

^f^Across-family,population-wide mean effect of the QTL peak marker, based on 200 cross-validation runs. Positive and negative signs indicate a trait-increasing and trait-decreasing effect of the wild barley allele compared to the Barke control allele, respectively. Values within the brackets show the effect estimates back-transformed to the original scale.

^g^Previously described stripe rust (*Psh*) and leaf rust (*Ph*) resistance QTL/genes located within the range of LD decay around the QTL marker with DR >50% identified in this study.

In both NAM studies a broad variation in the wild allele effect estimates of adjacent markers was observed (Fig 2; S6 File). Most of the QTL detected are composed of markers exhibiting opposed wild allele effect estimates, sometimes this holds true even for adjacent markers (S6 File).

The *Psh* resistance QTL showing the peak marker with the highest DR (i_SCRI_RS_209285) is located on the long arm of chromosome 3H. This SNP shows a negative cross-validated mean effect, resembling a decrease of the AO value in the presence of the wild allele compared to the Barke control allele (Fig 2; Table 2). In the *Ph* NAM study, the peak marker with the highest DR (i_SCRI_RS_184395) is located on the short arm of chromosome 2H and shows a positive cross-validated mean effect, representing an increase of the AO value in the presence of the wild allele compared to the Barke control allele (Fig 2; Table 2).

Estimation of wild allele effect estimates of robust *Psh* QTL peak markers across the whole population resulted in only small cross-validated mean effect estimates. Thus, resembling only minor increases or decreases of AO values of genotypes in the presence of the wild allele compared to the Barke control allele. Likewise, analysis of population-wide R^2^ values of QTL peak markers resulted in only low to intermediate estimates in the majority of cases (Table 2). Log_10_ based population-wide wild allele effect estimates range from -0.35 to 0.41 When back-transformed to the original scale this represents a maximum change in the AO value of 2.57% in the presence of the wild allele compared to the Barke control allele. The explained variance of a single QTL peak marker (R^2^) ranged from 0.09 to 12.91% (Table 2; S6 File).

Similar observations were made in case of *Ph*. Across the whole population robust *Ph* QTL peak markers showed only small wild allele effect estimates and low R^2^ values (Table 2). Log_10_ based population-wide wild allele effect estimates ranged from -0.28 to 0.30. When back-transformed to the original scale this represents a maximum change in the AO value of 2% in the presence of the wild allele compared to the Barke control allele. In case of the *Ph*. The explained variance of a single QTL peak marker (R^2^) ranged from 0.08 to 7.67% (Table 2; S6 File).

The peak markers of QPs.3H-4 and QPh.6H-2 showed the highest effect estimate (0.41 and 0.30; log_10_-value) and the peak markers of QPs.5H-1 and QPh.4H-1 the highest R^2^ value (12.91% and 7.67%) for trait AO_*Psh*_ and AO_*Ph*_, respectively (Table 2; S6 File).

After testing for QTL effects across HEB families, parent-specific QTL effects were calculated to obtain an effect estimate representing the combined effect of all family specific markers the QTL is composed of. Due to previously mentioned model limitations (see Material and methods), QTL QPs.3H-2, QPs.3H-3, and QPs.3H-4 were combined to one single parent-specific QTL QPs.3H-2/3/4, as well as QPs.5H-1 and QPs.5H-2 to one single parent-specific QTL QPs.5H-1/2.

For *Psh* as well as *Ph*, data estimation of parent-specific QTL effects revealed considerable variation in the effect size as well as direction of the wild allele between families (S7 File). In case of *Psh* only three parent-specific QTL showed the same effect direction across all families (QPs.2H-2; QPs.3H-1, and QPs.5H-1/2), whereas this was the case for five of the parent-specific QTL identified in the NAM study based on *Ph* data (QPh.2H-1, QPh.4H-2, QPh.5H-1, QPh.6H-1, and QPh.7H-3). No family showed trait-reducing effects at all parent-specific QTL, neither for *Psh* nor for *Ph*. The maximum count of parent-specific QTL showing a trait-reducing effect per family was five out of nine in case of AO_*Psh*_ and eight out of 11 for AO_*Ph*_. For trait AO_*Psh*_ family F24 (-0.01; log_10_-based value) and for trait AO_*Ph*_ family F03 (-0.54; log_10_-based value) showed the largest reducing effect summed up over all parent-specific QTL (S7 File). Results of the *Psh* and *Ph* NAM study revealed divergence between the QTL peak marker effect and the mean QTL effect based on the parent-specific QTL effects. For both traits QTL peak markers exhibited in most cases the same effect direction as the mean QTL estimate across HEB families, but differed in effect size (S7 File). Thus, NAM showed that QTL mean effect and peak marker effect are not necessarily identical in HEB-25.

The mean percentage of phenotypic variance explained through the full model (R^2^_adj_) was calculated to be 73.5% for *Psh* and 62.6% for *Ph*, respectively (Table 3). Notably, in case of both NAM studies a considerable portion of the phenotypic variance is explained by the robust QTL peak markers (Table 2; S6 File). The predictive ability (R^2^_pred_) of the full model for infection severity was calculated to be 42.4% for *Psh* and 32.3% for *Ph* (Table 3).

**Table 3:** Number of QTL and total phenotypic variance explained.

Trait^a^	QTL^b^	R^2^_adj_ (%)^c^	R^2^_pred_ (%)^d^
AO_*Psh*_	12	73.5	42.4
AO_*Ph*_	11	62.6	32.3

^a^Average ordinate for stripe rust (AO_*Psh*_) and leaf rust (AO_*Ph*_), respectively.

^b^Number of QTL defined for the respective trait.

^c^Mean phenotypic variance explained by the full NAM model.

^d^Mean ability to predict rust infection severity of independent genotypes.

### Comparison with previously identified QTL

Comparison of *Ph* resistance QTL identified in this study with those already reported in literature revealed that the majority of identified QTL mapped to chromosome regions known to be linked to *Ph* resistance. Nine out of the 11 QTL identified in this study conferring resistance to *Ph* showed overlap with marker intervals of previously reported *Ph* resistance QTL or genes (Table 2). Based on available data, LD based QTL intervals of QTL QPh.5H-1 and QPh.7H-2 showed no overlap with previously reported *Ph* resistance QTL or genes. In case of *Psh*, less overlap of resistance QTL identified in this study with those already reported was observed. Only four out of the 12 *Psh* resistance QTL identified in this study, namely QPs.3H-2, QPs.5H-1, QPs.6H-1, and QPs.7H-1, overlapped with previously reported *Psh* resistance QTL (Table 2). Four out of ten so far unknown resistance QTL for *Psh* or *Ph*, namely QPh.7H-2, QPs.2H-1, QPs.3H-3, and QPs.5H-2, showed negative CV mean effects (Table 2), indicating the existence of wild barley alleles conferring *Ph* or *Psh* resistance. The alignment of SNPs with DR >50% against the physical barley map by means of the BARLEYMAP pipeline resulted in the identification of a number of genes related to plant defence in the respective QTL intervals. In particular, leucine-rich repeat, NB-ARC, and serine/threonine-protein kinase-like domain genes were found at high frequency. Details are given in S2 File.

## Discussion

The strong variation in infection severity of HEB-25 lines infected with *Psh* and *Ph* in field trials demonstrates the high genetic diversity present within the HEB-25 population, and thus, its suitability to identify resistance QTL using NAM. Results of this study are in agreement with results of previous HEB-25 NAM studies that identified a comparable variation regarding developmental traits [69, 70] and salinity tolerance [71]. As in case of the previous HEB-25 studies, variation in *Psh* and *Ph* infection severity was detected between as well as within families, clearly indicating the suitability of HEB-25 to not only identify population-wide but also parent-specific QTL effects for resistance to *Psh* and *Ph* (Fig 1A and 1B; S3 File). The high variation in HEB-25 regarding stripe and leaf rust resistance is expected to be a function of the difference in the genetic make-up in the elite parent Barke and the wild donor parents. While wild donors showed in general a higher susceptibility to *Psh* than to *Ph* the opposite was true for the recurrent parent Barke (S5 File).

The evaluation of pathogen resistance in separate field trials for *Psh* and *Ph* allowed the individual phenotypic evaluation of HEB-25 genotypes without a potential bias caused by simultaneous infection of genotypes with both fungi. The integration of susceptible spreader strips and the inoculation of these with aggressive *Psh* and *Ph* isolates proved to be efficient as it allowed a clear and reliable differentiation of genotypes that would have been difficult to achieve under natural infection. This is particularly true for field trials conducted to identify QTL conferring resistance to *Psh*, as this fungus is strongly influenced by environmental conditions. We assume that the relatively high broad sense heritabilities (Table 1), which are a prerequisite for successful QTL identification, would not have been observed if phenotyping had been conducted based on natural infection.

The comparison of phenotypic results of the two field trials facilitated the identification of HEB lines that simultaneously showed a high degree of resistance against *Psh* and *Ph*. Out of these, especially HEB_03_006, HEB_03_015, and HEB_03_142 are valuable candidates to be integrated into barley pre-breeding programs aiming to simultaneously increase *Psh* and *Ph* resistance, as they are among the top one percent of genotypes regarding resistance to both pathogens (S3 File). However, next to a high level of resistance, results of earlier studies by Maurer et al. [69, 70] may be considered during the selection process to select resistant HEB lines, which combine a suitable resistance with elevated yield parameters. It is expected that the integration of favorable wild barley alleles into barley breeding programs will be achieved faster with HEB-25 lines than with wild barley accessions since a backcrossing step with cultivar Barke was already performed during the development of HEB-25.

The occurrence of opposed wild allele effect estimates of closely linked markers identified in this study was also observed in previous HEB-25 studies by Maurer et al. [69, 70, 72] and most likely arises from the fact that not all SNPs segregate in all families. Therefore, markers are likely to reflect only the mean wild allele effect of a fraction of the full population. As a result, closely linked markers segregating in different sets of genotypes of the complete population can show opposed effect estimates because of different mean resistance levels of the two sets. Phenotypic results revealed that families differ in their mean resistance level (Fig 1). Therefore, we assumed that strongly differing sets are likely to be linked to different families and, thus, opposed effect estimates of closely linked SNPs can be caused by parent-specific alleles.

Since the focus of this study was to identify robust QTL conferring resistance to *Psh* and *Ph* a rather stringent threshold for the acceptance of marker trait associations was defined. Minor QTL not passing this threshold but still influencing genotype response to *Psh* and *Ph* are not considered in this study. Defining a less stringent DR threshold of 10%, as applied in the study of Maurer et al. [70], would have resulted in a considerable higher number of individual QTL and co-locating QTL for the two rust fungi (Fig 2; S6 File). However, it has to be considered that with a lower DR threshold the risk of false-positive marker trait associations increases and, therefore, these minor QTL should be interpreted with caution.

The detection of QTL for resistance against *Psh* and *Ph* despite low estimates across the whole population is a strong proof of the power of the NAM strategy in general and in particular the suitability and precision of the NAM model applied in this study (Table 2; S6 File). The mean phenotypic variance explained by the full model and the calculated mean ability to predict the degree of infection of independent genotypes further supports the suitability of the applied model (Table 3).

The high number of QTL linked to *Psh* and *Ph* resistance detected in this study, the small CV mean effect estimates, as well as, the low percentage of phenotypic variance explained by the majority of QTL peak markers indicate a complex inheritance of adult plant resistance for both pathogens (Table 2; S6 File). QPs.5H-1 with a R^2^ value of 12.91% is an exception to the generally low phenotypic variance explained by the majority of QTL peak markers and might indicate the presence of a major resistance gene. Although there may be few lines carrying a major resistance gene, results of this study show that resistance in HEB-25 is predominantly polygenic and is the result of the accumulation of numerous small effect loci with additive effects. Similar results are reported in studies with other NAM populations focusing on stem rust of wheat [67] and stem rust, stripe rust, and leaf rust of wheat [68]. In each case, a high number of QTL with small to medium effects were reported and the authors concluded the nature of resistance to be polygenic, with several loci acting additively. However, the maximum allele effect estimates in the study of Li et al. [68] are higher compared to this study. The same holds true for R^2^ values in both studies. It has to be considered that next to being the result of a complex polygenic inheritance of resistance, small population-wide effects of QTL peak markers may also be attributed to the presence of alleles with differing effects on resistance. Namely, in case only a limited number of HEB-25 lines of the full population show a strong allele effect on resistance or contrasting allele effects among the 25 HEB donor parents exist at a marker position.

The importance of considering the influence of differing donor allele effects in HEB-25 on estimated population-wide QTL peak marker effects is supported by the high variation of donor allele effects at parent-specific QTL (S7 File). Results of this study are very similar to the observations made by Bajgain et al. [67] and Li et al. [68] focusing on the identification of QTL conferring resistance to rust pathogens of wheat by use of the NAM approach. As in this study the authors identified strongly varying parent-specific allele effects at resistance QTL. Therefore, studies focusing on detailed analysis of specific QTL or on the integration of *Psh* and *Ph* resistance alleles in modern barley cultivars should take into account the parent-specific QTL effect information given in this study to select the most promising resistance-carrying HEB line to be incorporated into a new barley breeding cycle. Not including parent-specific QTL effect estimates in the selection decision may result in missing alleles whose strong favorable effect is masked by a high number of parent-specific alleles with an opposed effect (S7 File). However, it is noteworthy to mention that parent-specific QTL effect estimates may be slightly biased, as each family comprises only a relatively small number of HEB-25 lines [72]. Thus, selection decisions should be based on a combined evaluation of population-wide and parent-specific estimates of wild allele effects.

Most *Ph* resistance QTL and several of the *Psh* resistance QTL identified in this study showed overlap with QTL previously reported to be linked to *Ph* or *Psh* resistance (Table 2). At the same time two QTL for resistance to *Ph* and eight QTL for resistance to *P*.*s* identified in this study are located at chromosome positions not yet reported to be involved in resistance against *Ph* or *Psh*, respectively. Several of the *Ph* and *Psh* resistance QTL, although showing no overlap with previously reported *Ph* and *Psh* resistance QTL, were located in chromosome regions known to be linked to resistance to *Ph* or *Psh*. In case of *Ph*, resistance QTL QPh.7H-2 is located in the vicinity of leaf rust resistance QTL Rphq8 identified by Qi et al. [27] and Marcel et al. [26] as well as QRph.sun-7H identified by Singh et al. [37]. Likewise, the resistance QTL QPs.1H-1 and QPs.2H-3 identified in the *Psh* NAM study are located in a chromosome region in which Dracatos et al. [44] identified a QTL linked to *Psh* resistance. Furthermore, QTL QPh.7H-2, QPs.3H-1, and QPs.3H-4 each show overlap with a meta-QTL identified by Schweizer and Stein [94] effective against several fungal barley pathogens. Based on available data and the QTL intervals defined in this study, all of the 10 QTL identified in this study to show no overlap (Table 2) should be regarded as potentially novel resistance QTL, harboring new and yet undiscovered rust resistance genes. It has to be considered, that the majority of previously reported *Ph* and *Psh* QTL were identified using different rust isolates than those used in this study. Therefore, QTL identified in this study showing overlap with previously reported *Ph* or *Psh* resistance QTL may potentially confer novel resistance alleles at known rust resistance loci.

Only at three chromosomal locations, QTL for stripe rust and leaf rust resistance co-localized. This finding may indicate the existence of rust specific defense mechanisms in HEB-25 rather than a broad-spectrum species-independent pathogen control. This assumption is also supported by a low correlation observed between both traits. The clear preponderance of independent QTL in HEB-25, either specific for *Ph* or *Psh*, is in agreement with Suenaga et al. [95]. The authors detected only one common QTL for leaf rust and stripe rust resistance in wheat. In contrast, studies by McIntosh [96] and William et al. [97] showed correlated response of wheat to leaf rust and stripe rust caused by closely linked genes. Likewise, Herrera-Foessel et al. [98] observed a correlated response to leaf rust and stripe rust of wheat for most of the tested wheat lines caused by either a single gene or very closely linked genes conferring resistance to both pathogens. Furthermore, Krattinger et al. [99] identified *Lr34*, a broad-spectrum non-race-specific resistance gene that confers resistance to a range of pathogens including leaf rust and stripe rust of wheat. Next to this, William et al. [100] and Li et al. [68] both reported QTL conferring resistance to leaf rust and stripe rust of wheat as well as QTL conferring resistance to only one of the two pathogens. The *Ph* and *Psh* resistance QTL located in close proximity to each other (QPs.2H-1 and QPh.2H-1, QPs.6H-1 and QPh.6H-2, QPs.7H-1 and QPh.7H-3), may represent regions linked to general resistance to rust fungi, and thus, be combined to meta-QTL. However, peak markers of co-locating *Ph* and *Psh* resistance QTL in HEB-25 showed opposed wild allele effects (Table 2). This fact points towards the presence of two independent rust pathogen specific resistance genes located in proximity to each other, rather than the presence of a single resistance gene conferring resistance to both pathogens. The fact that the three co-localized *Ph* and *Psh* resistance QTL were not identified at the same chromosomal position, but were located within a distance of 2.5 (2H) to 9.0 (7H) to each other further supports this assumption.

We found a high frequency of leucine-rich repeat, NB-ARC, and serine/threonine-protein kinase-like genes as putative candidate genes in rust resistance QTL intervals of HEB-25. This finding is in agreement with the important role of those gene families, known as resistance gene analogs (RGAs), in various defence reactions of plants against pathogens [101, 102]. Based on this study the definition of a single candidate gene responsible for the detected QTL effect is not feasible. The final prove, which candidate gene is causing the QTL effect, may be achieved after a high-resolution mapping within the respective QTL interval has been conducted and the identified candidate genes, co-segregating with the resistance phenotype, have been knocked-out or genetically engineered. The various putative candidate genes identified in this study by the use of the BARLEYMAP pipeline and GO-term analysis within the QTL intervals may serve as a starting point for subsequent studies focusing on the genetic basis of resistance of barley to *Ph* and *Psh* (S2 File).

## Conclusion

The results of this study provide valuable information not only for basic studies elucidating the molecular basis of *Psh* and *Ph* resistance in barley, but also for improving *Psh* and *Ph* resistance and diversity of modern elite barley cultivars. We expect that in future a better understanding of the allelic diversity present at stripe rust and leaf rust QTL in HEB-25 will be achieved by generating exome capture based SNP and haplotype data for all HEB lines and 26 HEB parents. This way, it is expected to achieve more precise estimates of haplotype-based allele effects in HEB-25 and to increase the power to detect wild barley alleles with favorable effects on barley resistance against stripe rust and leaf rust.

## Supporting information

S1 File LD decay of intra-chromosomal markers across HEB-25. (PDF)

S2 File Alignment of markers with DR >50% using BARLEYMAP. (XLSX)

S3 File Two-year least squares means (lsmeans) of HEB lines for traits AOPsh and AOPh (i.e. stripe and leaf rust symptoms, respectively). (XLSX)

S4 File Frequency distribution of two-year lsmeans for trait for traits AOPsh and AOPh (i.e. stripe and leaf rust symptoms, respectively). (PDF)

S5 File Average ordinate (AO) values of HEB-25 parents. (PDF)

S6 File GWAS results for resistance against strip rust (*Psh*) and leaf rust (*Ph*) in HEB-25. (XLSX)

S7 File Estimates of parent-specific QTL effects and QTL mean effects across HEB families for or traits AOPsh and AOPh (i.e. stripe and leaf rust symptoms, respectively). (XLSX)
