# Supplementary material for: Identification of QTL conferring resistance to stripe rust (Puccinia striiformis f. sp. hordei) and leaf rust (Puccinia hordei) in barley using nested association mapping (NAM)
Source: PLoS One. 2018 Jan 25;13(1):e0191666. doi: 10.1371/journal.pone.0191666 (PMC5784946; doi:10.1371/journal.pone.0191666)
Supplement: S1 File — (PDF) [file pone.0191666.s001.pdf]

Supporting Information 1

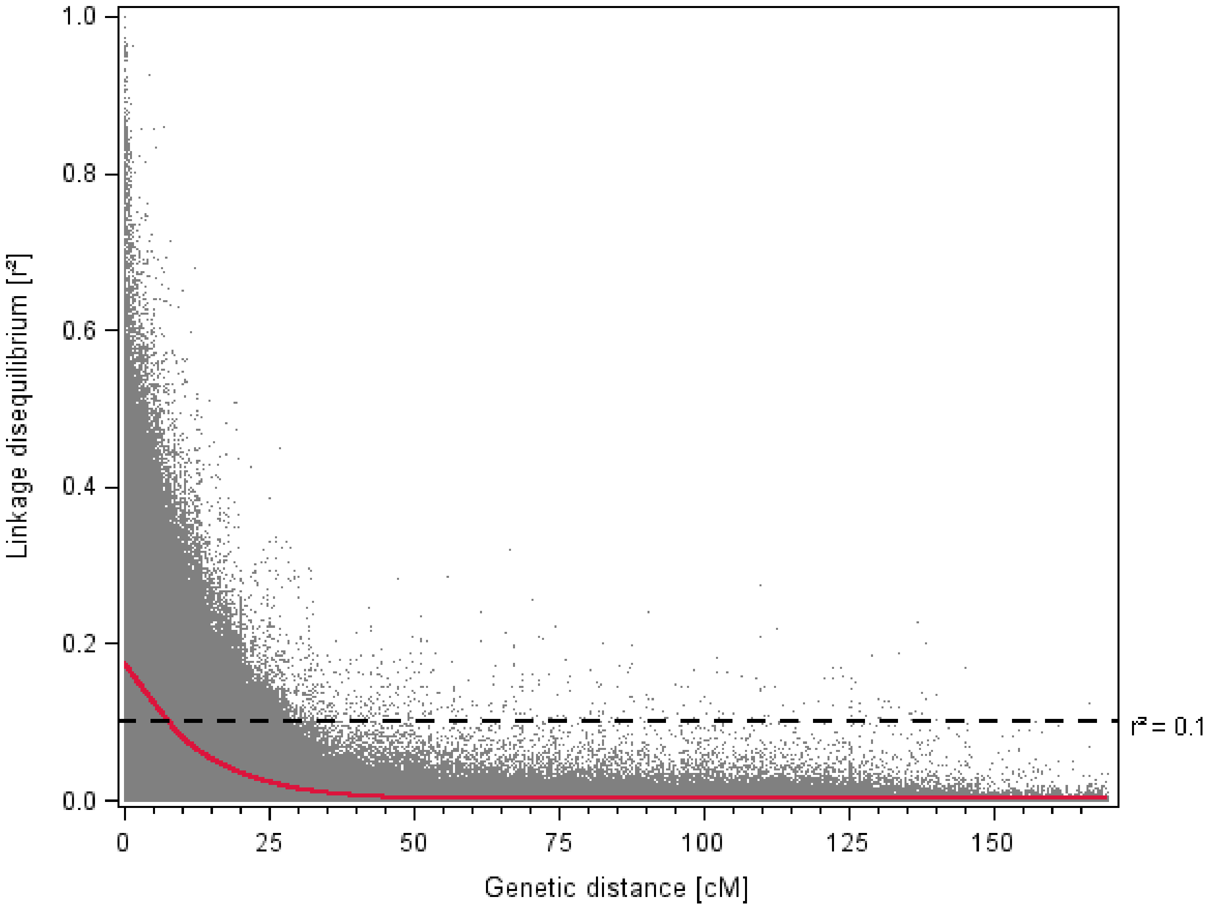

LD decay of intra-chromosomal markers across HEB-25. The red curve and the dashed black line indicate the second degree loess fit and the threshold of LD, based on the 95th percentile of inter-chromosomal SNPs, respectively. LD decay, defined as the distance where the loess curve crosses the threshold, is 7.85 cM.
