# Supplementary material for: Identification of QTL conferring resistance to stripe rust (Puccinia striiformis f. sp. hordei) and leaf rust (Puccinia hordei) in barley using nested association mapping (NAM)
Source: PLoS One. 2018 Jan 25;13(1):e0191666. doi: 10.1371/journal.pone.0191666 (PMC5784946; doi:10.1371/journal.pone.0191666)
Supplement: S4 File — (PDF) [file pone.0191666.s004.pdf]

Supporting Information 4

A

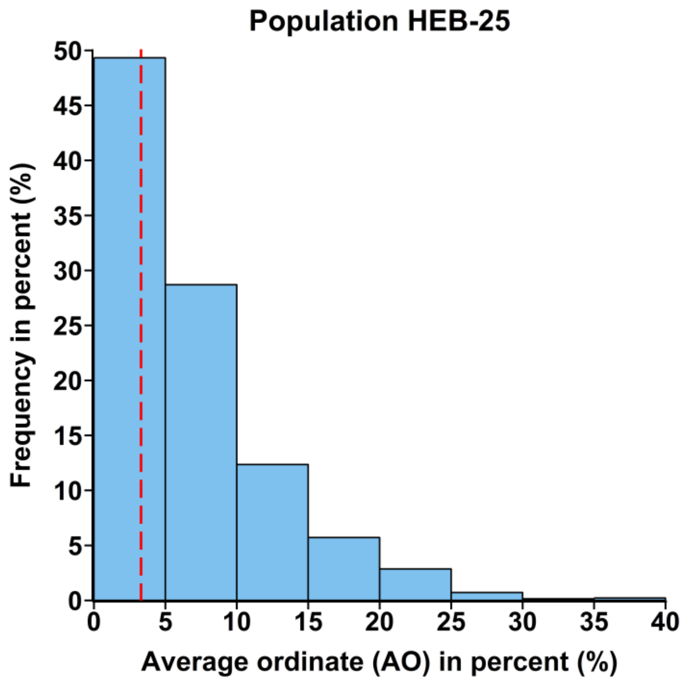

B

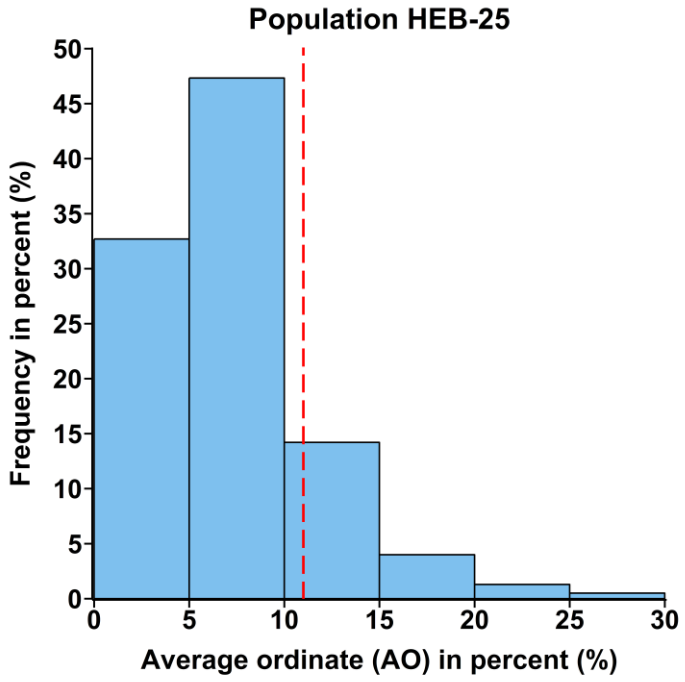

Frequency distribution of two-year average ordinate (AO) values for **(A)** stripe rust (AO*P.s.*), and **(B)** leaf rust (AO*P.h.*). The dotted red line depicts the lsmeans value of the recurrent parent Barke for the respective trait.
