# Supplementary figures and images for: Identification of QTL conferring resistance to stripe rust (Puccinia striiformis f. sp. hordei) and leaf rust (Puccinia hordei) in barley using nested association mapping (NAM)
Source: PLoS One. 2018 Jan 25;13(1):e0191666. doi: 10.1371/journal.pone.0191666 (PMC5784946; doi:10.1371/journal.pone.0191666)

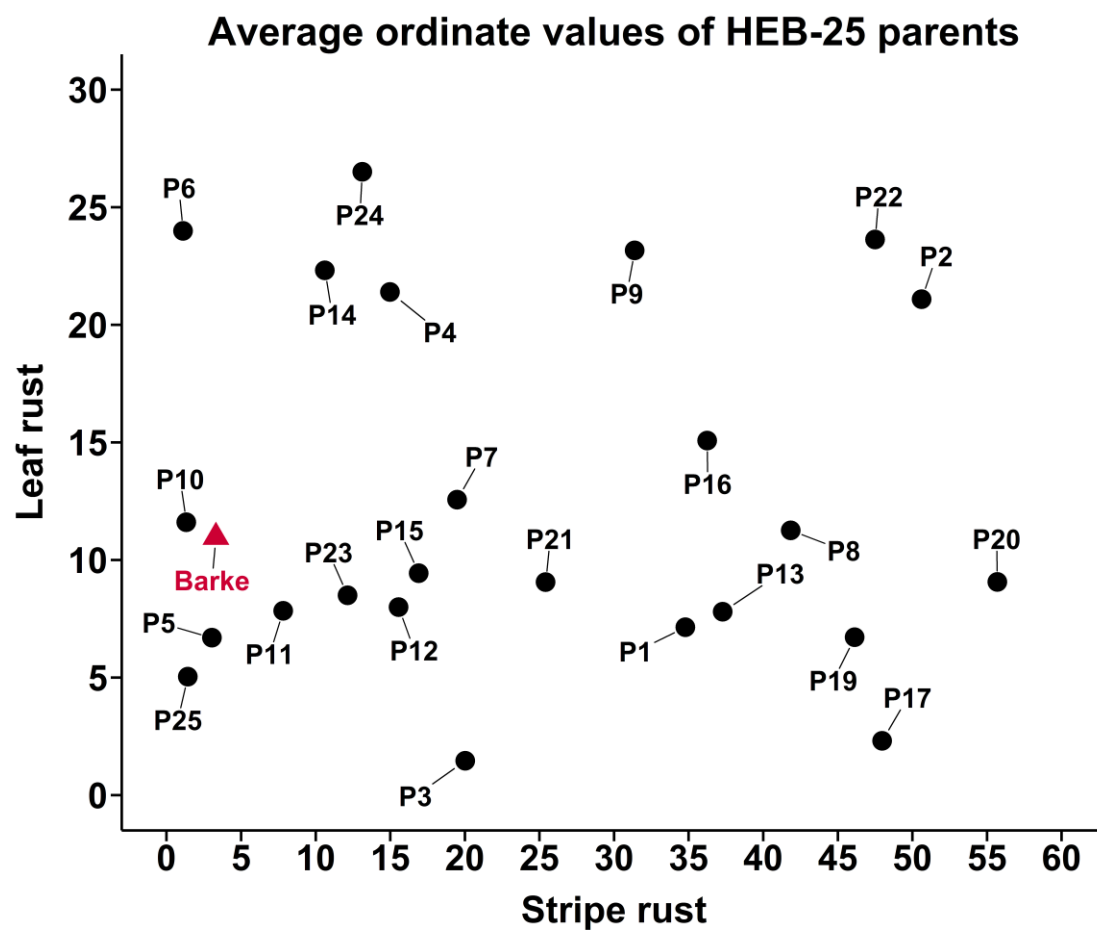

Average ordinate (AO) values of the wild donor parents of HEB-25 for stripe rust (*P.s.*) and leaf rust (*P.h.*).

Supplement: S5 File — (PDF) [file pone.0191666.s005.pdf]
